# PLDLA/TPU Matrix Enriched with Cyclosporine A as a Therapeutic Platform for Immune-Mediated Keratitis (IMMK) in Horses

**DOI:** 10.3390/ijms24065735

**Published:** 2023-03-17

**Authors:** Martyna Padjasek, Anna Cisło-Sankowska, Anna Lis-Bartos, Badr Qasem, Krzysztof Marycz

**Affiliations:** 1 Department of Experimental Biology, The Faculty of Biology and Animal Science, The University of Environmental and Life Sciences, 50-375 Wroclaw, Poland; 2 International Institute of Translational Medicine, Jesionowa 11 St., 55-124 Malin, Poland; 3 Department of Biomaterials and Composites, Faculty of Material Science and Ceramics, AGH University of Science and Technology, Aleja Adama Mickiewicza 30, 30-059 Krakow, Poland

**Keywords:** poly (L-lactide-co-DL-lactide), thermoplastic polyurethane, cyclosporin A, immune-mediated keratitis

## Abstract

The purpose of this study was to describe the use of PLDLA/TPU matrix enriched with cyclosporine A (CsA) as a therapeutic platform in horses with immune-mediated keratitis (IMMK) with an in vitro evaluation CsA release and degradation of the blend as well as determination of the safety and efficacy of that platform used in the animal model. The kinetics of the CsA release from matrices constructed of thermoplastic polyurethane (TPU) polymer and a copolymer of L-lactide with DL-lactide (PLDLA) (80:20) in the TPU (10%) and a PLDL (90%) polymer blend were studied. Moreover, we used the STF (Simulated Tear Fluid) at 37 °C as a biological environment to assess the CsA release and its degradation. Additionally, the platform described above was injected subconjunctival in the dorsolateral quadrant of the globe after standing sedation of horses with diagnosed superficial and mid-stromal IMMK. The obtained results indicated that the CsA release rate in the fifth week of the study increased significantly by the value of 0.3% compared to previous weeks. In all of the cases, the TPU/PLA doped with 12 mg of the CsA platform effectively reduced the clinical symptoms of keratitis, leading to the complete remission of the corneal opacity and infiltration four weeks post-injection. The results from this study showed that the PLDLA/TPU matrix enriched with the CsA platform was well tolerated by the equine model and effective in treating superficial and mid-stromal IMMK.

## 1. Introduction

Immune-mediated keratitis (IMMK) in horses is a nonulcerative, primary keratitis [1], characterized by chronic (lasting more than three months) corneal opacities [2]. Along with the visible opacity of the cornea, vascularization, cellular infiltration, and corneal edema are observed [3]. The pathology could be manifested bilaterally but it is more frequently reported as unilateral [3]. Horses with IMMK show mild or no signs of ocular discomfort like slight epiphora and blepharospasm. Histopathological examination reveals mostly lymphocytic-plasmacytic cells and in some cases, neutrophils, and macrophages [3,4]. Furthermore, immunopathology studies suggest a t cell-driven inflammation without a significantly greater extent of CD4+ or CD8+ [3,5]. Despite numerous studies, the exact etiology and pathophysiology are still unknown but due to cellular findings and good response to immunomodulatory and anti-inflammatory therapy, suggesting immune-mediated or autoimmune processes [2,5]. Therefore, the main diagnosis is based on clinical presentation, an absence of infectious agents and a good response to medication [6]. The clinical appearance depends on the lesion location in the cornea and the type of cells involved in infiltration. The USA distinguishes five types of IMMK—epithelial, superficial stromal, mid-stromal, endothelial, and eosinophilic keratitis in which the main cells are eosinophils. Despite different descriptions and clinical presentation (i.e., chronic superficial keratitis, chronic recurrent keratitis, endotheliitis, and epithelial keratopathy) the UK cases also meet the diagnostic criteria of IMMK [2]. The latest study with confocal microscopy (IVCM) showed that in cases of epithelial, superficial stromal, and mid-stromal IMMK, a characteristic feature was dendritic cells mainly located in the epithelial basement membrane and subepithelial stroma, which was not observed in endothelial IMMK or another equine keratitis [4]. Due to the suspicion of autoimmune causes, the therapy is based on anti-inflammatory and immunosuppressive medications and depends on the location of the inflammatory lesion [7]. In some cases, an inadequate response to local treatment necessitates surgical intervention—keratectomy and a conjunctival graft [6]. The candidate drug of choice in equine keratitis is CsA [8,9].

CsA is a lipophilic, cyclic undecapeptide weighing 1202.6 g/mol MW, with poor solubility in water and limited bioavailability [10]. As a drug, it is used in human and veterinary medicine transplantation procedures as well as the broad spectrum of immune-mediated inflammatory diseases (IMIDs) due to its strong immunosuppressive potency [11,12]. However, due to cyclosporine’s physicochemical properties, its administration may cause several side effects including hepatotoxic, cardiotoxic, nephrotoxic, and neurotoxic effects [12] Therefore, new cyclosporine A sustained delivery implants in veterinary ophthalmology are essential to ensure high bioavailability and long-term release with minimally invasive implantation procedures, especially in non-cooperative horses with less committed owners [7,9,13,14,15,16].

Previous studies have proposed the use of a silicone matrix CsA episcleral implant in horses with different types of immune-mediated keratitis [7], demonstrating the effective application of a sustained-release CsA implant in this disease.

This study proposes and describes the successful use of a TPU/PLDLA polymer blend containing CsA to manage superficial and mid-stromal immune-mediated keratitis in horses.

## 2. Results

### 2.1. Dynamic Contact Angle Measurement of TPU/PLDLA Blends

**Figure 1:**
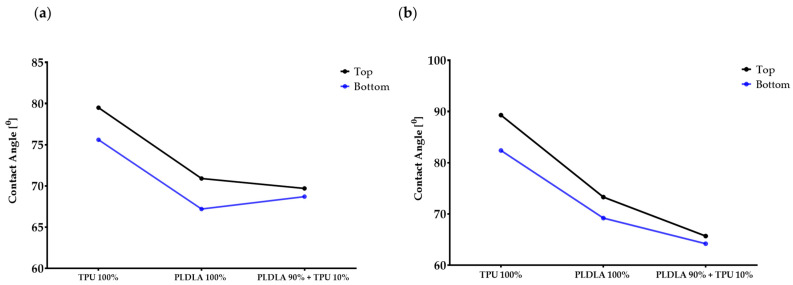
The contact angle measurements of the water and Diiodomethane dropped on polystyrene Petri dishes with TPU/PLA and TPU/PLA/CsA films. (**a**); water contact angle, (**b**); diiodomethane contact angle.

To identify any alterations in the behavior of the TPU/PLDLA blends that can affect the CsA release, the hydrophobicity characteristic of polymer films was investigated in both water and diiodomethane dropped using a contact angle analyzer then the average of ten times’ measurement was calculated (Table 1). The results showed the water contact angle on the bottom surface was lower than the top surface and the same trend was noticed with the diiodomethane contact angles for all film surfaces (Table 1). Generally, the water contact angle of the TPU/PLDLA blend was higher than the diiodomethane contact angle and showed overall decreased hydrophobicity compared to pure TPU/PLDLA films (Table 1, Figure 1).

### 2.2. The In Vitro Degradation Analysis

The degradation rate of TPU/PLDLA films and blends was assessed in vitro by measuring the changes in the mass percentage and pH value at 37 °C. These measurements serve as indicators of the rate and extent of polymer degradation. TPU/PLDLA is a degradable material that undergoes hydrolysis, leading to the release of acidic degradation products such as lactic and glycolic acid. Consequently, the pH of the surrounding STF medium may decrease due to the accumulation of these acidic degradation products.

**Figure 2:**
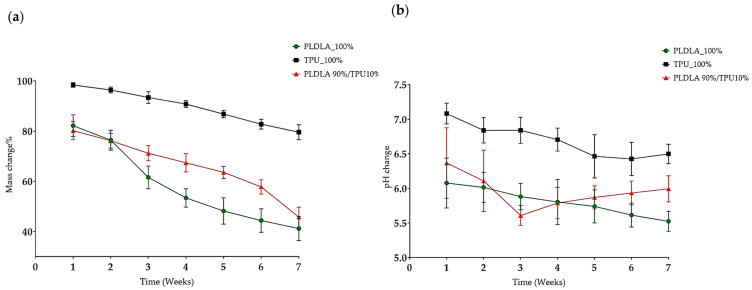
The mass and pH changes of the polymers PLDLA/TPU films and PLDL + TPU blend over 7 weeks incubated in simulated tear fluid (STF). (**a**); The mass changes; (**b**); The pH changes. Data points in the line plots were calculated based on the mean values of five replicates for each week interval with standard deviation error bars. In the boxplot: whiskers = 1.5 × IQR, lines = average; boxes = interquartile range Q1–Q3.

The mass percentage of each material was measured at a neutral pH of 7.4, as well as the pH of the solution. In general, it can be observed that the mass percentage of both PLDLA and TPU decreases over time, while the mass percentage of the TPU/PLDLA combination increases. During week 1, the mass percentage of PLDLA was 82.2%, while the mass percentage of TPU was 98.4%, and the TPU/PLDLA combination was at 80.2%. The pH of the TPU was 7.08, which was slightly higher than the pH of the PLDLA at 6.08. The pH of the TPU/PLDLA combination was 6.37. Over the following weeks, it can be observed that the mass percentage of each material decreased gradually, with the TPU/PLDLA combination showing the least amount of degradation. The pH remained relatively constant throughout the study period, indicating that there was no significant chemical breakdown of the materials or the combination (Table 2, Figure 2).

### 2.3. The CsA In Vitro Release Analysis

**Figure 3:**
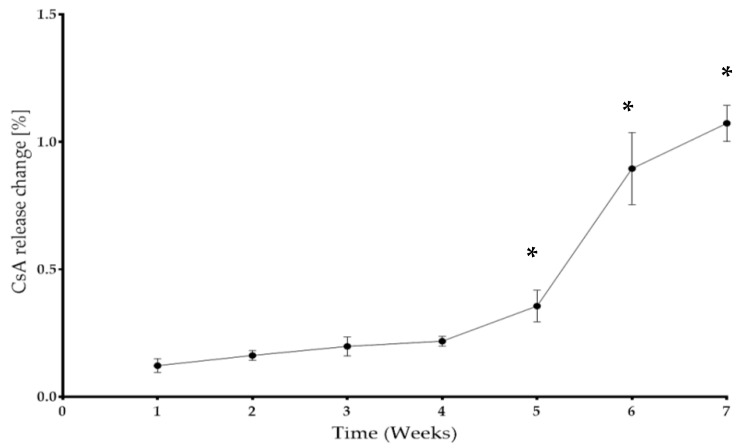
The cyclosporin A released from the matrix (PLDLA 90% + TPU 10%) over 7 weeks. Data points in the line plots were calculated based on the mean values of five replicates for each week interval with standard deviation error bars. In the boxplot: whiskers = 1.5 × IQR, lines = average; boxes = interquartile range Q1–Q3. * Adjusted *p* value < 0.05 and significant difference from all groups.

The CSA release profile from the matrices made of a 90% PLDLA and 10% TPU blend was assessed to determine the degradation time of the CsA implants and the kinetics of the immunosuppressive drug release (Table 3, Figure 3). In the first week, the release of CSA was observed at the level of less than 0.2%, then for the next 4 weeks (in the fifth week) the amount of cyclosporine A released increased significantly; it exceeded the value of 0.3%. In the sixth and seventh weeks, there is a significant increase in the amount of CSA released compared to the previous weeks.

### 2.4. The Clinical Outcome 

**Figure 4:**
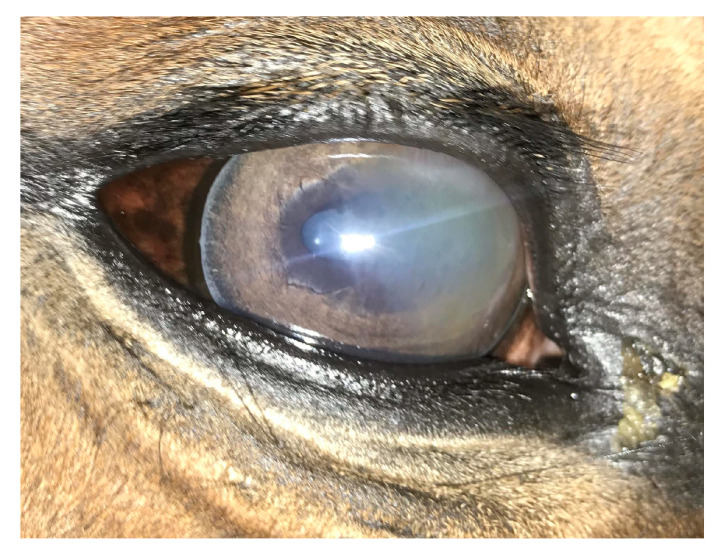
Diminish of blepharospasm and ocular discharge in case of a 6-year-old gelding of Polish Halfbred Horse.

**Figure 5:**
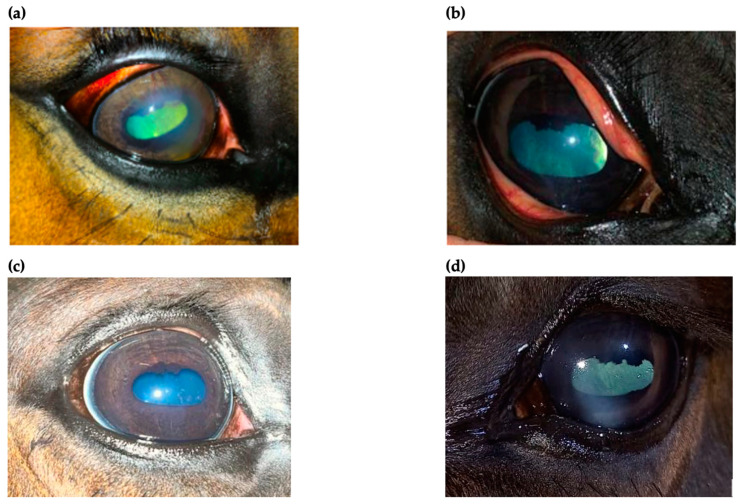
Superficial immune-mediated keratitis (IMMK) treated with TPU/PLDLA/CsA matrix implants 2 weeks post-injection (**a**,**d**) and mid-stromal IMMK with no corneal abnormalities detected after 6 weeks (**b**) and after 1 year (**c**).

An ophthalmic examination was performed at 24 h post-injection. Examination revealed no adverse effects after injection and no improvement in the clinical appearance as well. Re-check examinations were performed every 24 h for the next 2 weeks. There was no significant improvement in corneal appearance for the first week, but blepharospasm and discharge started to decrease gradually at 3 days post-injection (Figure 4). After 2 weeks post-injection, both ocular discharge and blepharospasm were absent. An ophthalmic examination revealed a significant decrease in corneal oedema with an absence of cellular infiltrate within the cornea. Perilimbal vascularization also diminished, and the normal size of the pupil was noticed (Figure 5a,d). No corneal abnormalities were detected after 6 weeks (Figure 5b). All of the horses with superficial keratitis responded well to implantation of the TPU/PLDLA/CsA matrix devices, and the IMMK was controlled. Six horses with mid-stromal IMMK showed a long-term clinical improvement. In one horse, there was no response to the treatment. Two horses presented for re-examination after six-months post-implantation revealed no recurrence of the disease and the last examination, performed after a year in the case of mid-stromal IMMK, still showed no signs of keratitis (Figure 5c).

## 3. Discussion

Immune-mediated keratitis is a nonulcerative, inflammatory corneal disease of horses, manifested with opacity, vascularization, edema, and cellular infiltrate of the cornea, that commonly occurs unilaterally and pain-free. Moreover, patients presenting IMMK usually show no signs of intraocular inflammation [1,2,4]. Further research is required to better understand the etiology and pathophysiology, thereby improving the treatment of all types of IMMK. Nowadays, the diagnosis is based among others on ophthalmologic examination and a good response to topical CsA.

Cyclosporine A is a calcineurin inhibitor (CNI), a selective immunosuppressant, which affects the activation process of T lymphocytes [17]. Due to specific properties, CsA finds an application in the therapy of immune-mediated diseases such as keratoconjunctivitis sicca (KCS) [18,19], chronic superficial keratitis (CSK) [20], equine recurrent uveitis (ERU) [21] and also immune-mediated keratitis (IMMK). Along with ERU, with a reported prevalence of up to 25% [22], IMMK is a common corneal disease that occurs in horses worldwide. Improper therapy can lead to secondary complications that affect the comfort of life, and cause vision impediment and even blindness [23]. The chronicity of the disease may be problematic for the owner, especially when the patient is uncooperative and the therapy requires the administration of drugs several times a day. That is why a sustained release CsA is a good solution to ensure the effectiveness of the therapy. Previous studies using a silicone matrix CsA episcleral implant showed the effective use of this type of therapy in the treatment of IMMK, especially superficial keratitis. However, the implantation of the devices required a surgical procedure under general anesthesia, and to achieve a therapeutic concentration of CsA, an application of 2–4 devices was required. In less developed countries, where special care is not widely available and appropriate surgical preparation and general anesthesia may be unavailable or too expensive, it is necessary to use procedures that are less invasive and easy to perform in most conditions. All these factors pushed us to propose a TPU/PLDLA polymer blend containing CsA as a therapeutic platform for IMMK.

The most common biocompatible, biodegradable nontoxic biomaterials used for medical applications are synthesized polymers including polylactide acid (PLA) [24], and polyurethane (PU) [25]. However, the PLA alone cannot be used, except in hard tissue scaffolding due to its high strength and brittle characteristics [26,27,28,29]. For that reason, several studies have been focusing on fabricating PLA blends with thermoplastic polyurethane (TPU) [30]. Here, we have found that the biodegradation time of the PLDLA/TPU matrix enriched with cyclosporine A released a high amount on the fifth day. Moreover, the PLDLA/TPU blends showed long-term effects from the degradation of their mass by 45.8% after 7 weeks.

Fifteen horses were included in the study with diagnosed superficial and mid-stromal IMMK. Clinical manifestations, such as the white-yellow corneal opacity, blepharospasm, and moderate discomfort were similar in all of the cases. An ophthalmological examination revealed mucopurulent discharge in the medial canthus, conjunctival hyperemia, corneal vascularization, and superficial or mid-stromal cellular infiltration of the cornea. Corneal cytology and culture confirmed the absence of microorganisms, whereas the histological examination was not performed due to the lack of consent of the owners. After discussing the therapeutic possibilities with the owners, the decision to apply a sustained-release CsA platform was made.

A simplified procedure enables implantation after standing sedation. Prior to subconjunctival injection, in the dorsolateral quadrant of the globe, an auriculopalpebral nerve block and topical anesthesia were performed. The clinical signs observed in this report decreased week by week, leading to a complete corneal translucency, without signs of inflammation 4 weeks post-implantation; likewise in cases of superficial keratitis and mid-stromal keratitis. In two horses, a re-examination after a minimum of six-months post implantation revealed no recurrence of the disease, confirming the clinical effectiveness of the TPU/PLDLA polymer blend containing CsA as a therapeutic platform for at least that period of time. The implants were well tolerated throughout the follow-up period and only a slight hematoma at the injection area was observed at the beginning. In the research group presented in this study, fourteen out of fifteen horses showed the satisfactory therapeutic effect and regression of the clinical symptoms.

The main limitations of this study were the small number of horses included, representing different types of IMMK, no comparative control group, using a placebo implant and demonstrating the effect of the polymer blend in long-term, which was not possible in this study due to client-owned horses, a lack of histopathological samples and a short follow-up time; therefore, further study is required. However, in vitro research and clinical outcomes give a positive perspective for the future. An ICVM study found that anti-inflammatory and immunosuppressive medications failed to reach and eliminate the population of dendritic cells within the cornea and suggest that keratectomy, which aims to remove the diseased layers of the cornea, combined with an anti-inflammatory and immunosuppressive treatment may bring the sufficient means of controlling IMMK [4]. ICVM findings may be crucial in determining the effectiveness of the TPU/PLDLA polymer blend containing CsA in eliminating the dense network of dendritic cells in the cornea. This study is an introduction to further research work. Simplifying the surgical procedure is crucial to reaching more clinicians, therefore helping more horses with IMMK. Wide availability and an easy implantation procedure without the need for general anesthesia will allow a faster and more effective treatment, which may prevent secondary complications, including blindness.

## 4. Materials and Methods

### 4.1. Materials

The biocompatible and biodegradable TPU/PLDLA blend and TPU/PLDLA blends with cyclosporine A (CsA) content were tested. The elastomeric thermoplastic polyurethane TPU (Ellastolan^®^ A12P000-INTiBS) was purchased from the Polish Academy of Sciences (PAN) (Wroclaw, Poland). Rigid polylactide “PURASORB^®^” (PLA), with a molar ratio of 80% poly-L-lactide and 20% poly-DL-lactide was purchased from PURACbiochemby (Gorinchem, The Netherlands). Cyclosporine A was purchased from Sigma-Aldrich (Poznan, Poland).

### 4.2. Preparation of TPU/ PLDLA Blends

Matrices of biodegradable thermoplastic polyurethane and L-lactide and D,L-lactide copolymer (80:20) blends were prepared by dissolving the polymers in N,N-dimethylformamide (DMF) and dichloromethane (DCM) solvents, respectively. A polymer blend with a weight ratio of 90:10 for PLDLA and TPU with 12 mg of CsA was prepared. The blend with CsA solutions were prepared by dissolving the PLDLA and TPU polymers in a DMF/DCM mixture (50:50% volume ratio) with a total polymer concentration of 5% w/w. The mixture was stirred continuously with a magnetic stirrer in a glass bottle at 40 °C for 4 days until a homogeneous mixture solution was obtained. The solution was allowed to stand at room temperature in an air atmosphere for 1 day to eliminate any air bubbles.

### 4.3. Contact Angle Measurements

In order to evaluate the wettability of the sample and its hydrophobicity characteristic, drop shape analysis by DSA 10 Mk2 Goniometer (Kruss, Hamburg, Germany) was performed. The measurement was conducted using ultra-pure water from the PURE LAB UHQ Model apparatus (Vivendi Water Solution), and diiodomethane (Sigma-Aldrich, Poznan, Poland). The surface of the PLDLA/TPU/CsA blends was tested for droplet measurements using approximately 0.2 μL volume at 25 ± 0.5 °C. The measurements were performed ten times using different droplets.

### 4.4. In Vitro Release Test

The in vitro release of 12 mg of cyclosporine A was studied from a 0.4 mL TPU/PLDLA blend using a UV-VIS spectrophotometer in 100 mL simulated tear fluid (STF) maintained at 37 ± 1 °C. The simulated tear fluid (STF) (pH 7.4) was prepared as the dispersion medium in the in vitro release study. The composition of the STF was as follows: NaCl (0.68 g), NaHCO3 (0.22 g), CaCl2⋅2H2O (0.008 g), KCl (0.14 g), and distilled deionized water to a total volume of 100 mL, then the STF solution was filtered through a 0.2 μm pore size filter.

The TPU/PLDLA disc samples with a diameter of 14 ± 1 mm were placed in dissolution PP vessels and rotated at 500 rpm using a paddle. At weekly intervals, 10 mL of sample was withdrawn, filtered through a 0.45 μM pore size membrane filter, and measured for CsA concentration using a spectrophotometer.

### 4.5. In Vitro Degradation Measurements in STF

The in vitro degradation study of TPU/PLDLA and TPU/PLDLA (0.4 mL)/CsA (12 mg) was conducted in STF (Simulated Tear Fluid) at 37 °C.

The weighted polymer films were placed in 30 mL polypropylene (PP) tubes containing 20 mL simulated tear fluid and then incubated at 37 °C. for up to 7 weeks. At weekly intervals, the simulated tear fluid (STF) was replaced, and the concentration of the CSA drug was determined in the collected samples via UV-VIS spectroscopy at 202 nm. The mass of dried samples was measured by a WPS30 moisture analyzer (Radwag, Wroclaw, Poland) after incubation at 37 °C and the pH of the STF was also measured using a CP-411 pH meter (Elmetron, Zabrze, Poland), and then replaced with fresh STF.

### 4.6. Evaluation of the Records of Horses Diagnosed with IMMK

**Figure 6:**
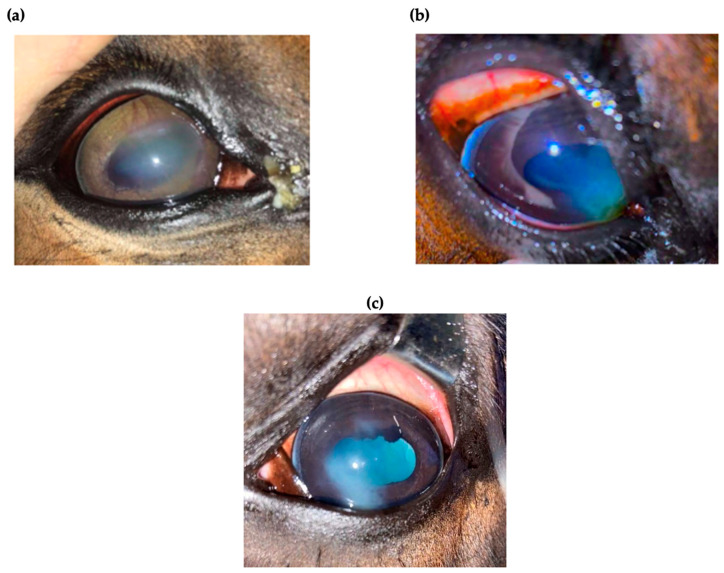
Images represent the two cases of immune-mediated keratitis (IMMK). (**a**) Mucopurulent discharge, mild conjunctival hyperemia, superficial vascularization corneal opacity with cellular infiltration within the mid stroma of the cornea. (**b**) Conjunctival hyperemia, mild mucus discharge and cellular infiltration within the superficial and mid stroma of the cornea. (**c**) Mild conjunctival hyperaemia, corneal edema, and cellular infiltration within the mid stroma of the cornea.

This study was conducted by analyzing horses diagnosed with IMMK. Fifteen horses of different ages (range 6–15 years old), sexes, and breeds, diagnosed with IMMK, were selected for the study based on the characteristic signs in the ophthalmic examination, the absence of microorganisms in corneal cytology and culture, a satisfactory response to topical treatment, and difficulties of the owner in continuing long-term treatment. Briefly, horses were presented with typical for IMMK ocular signs, such as discharge, white-yellow opacity in nasal quadrants of the cornea, and corneal edema with mild signs of blepharospasm and discomfort. The anterior aspects of the eye were examined with the slit lamp (SL-17 Portable Slit Lamp, Kowa American Corporation, Torrance, CA, USA) and revealed some mucopurulent discharge in the medial canthus, mild conjunctival hyperemia, and corneal opacity with cellular infiltration within the superficial stroma or middle stroma of the cornea. Corneal perilimbal superficial vascularization was noticed. The pupil was slightly miotic in some cases (Figure 6a–c). A Schirmer tear test I (Tear Touch, Madhu Instruments PVT LTD, New Delhi, India) (STT I) revealed bilateral aqueous tear production on a normal reference range and intraocular pressure (IOP) was measured with a tonometer (Tono-Vet, Icare, Vantaa, Finland). Atropine (Atropinum sulfuricum WZF 1%, Polfa, Warszawa, Poland) eye drops were administered to both eyes to dilate the pupils. Then, using an indirect ophthalmoscope (Heine Omega 500 Binocular Indirect Ophthalmoscope, HEINE Optotechnik GmbH & Co. KG, Gilching, Deutschland) the intraocular examination was performed, which confirmed the absence of pathological intraocular changes, including fundus pathologies. Corneal cytology and culture confirmed the absence of microorganisms. Performing keratectomy in client-owned horses for histological examination was not possible due to the invasiveness of the procedure. Eight horses were classified as having superficial keratitis and seven of them as having mid-stromal IMMK. The long-term administration of CsA was recommended.

### 4.7. The Clinical Procedures

Due to the behavior and temperament of the horses, a decision was made to apply a sustained-release CsA implant in all of the cases. Horse owners were fully informed about this experimental procedure and signed a written agreement for the therapy.

**Figure 7:**
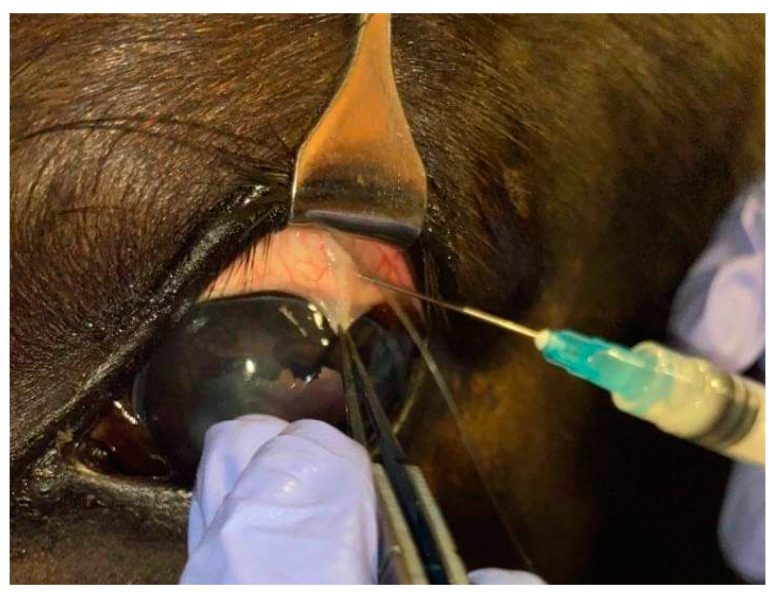
Performing a subconjunctival injection in the dorsolateral quadrant with placement of TPU/PLDLA implant containing CsA.

Standing sedation was performed with 10 μg/kg IV detomidine (Domosedan, Orion Corporation, Espoo, Finland) and 10 μg/kg IV butorphanol (Butomidor, Orion Corporation, Espoo, Finland). An auriculopalpebral nerve block was performed using 1–2 mL of lidocaine hydrochloride (Lignocainum hydrochloricum WZF 2%, Polfa, Warszawa, Poland), and proxymetacaine (Alcaine, Alcon Polska Sp.z o.o., Warszawa, Poland) was applied topically to the cornea. After sedation, a TPU/PLDLA implant containing CsA was implanted subconjunctival in the dorsolateral quadrant of the globe (Figure 7). Following the implantation, a slight hematoma at the injection area was observed.

### 4.8. Statistical Analysis

The dataset was tested by one-way ANOVA, Tukey’s multiple-comparison post test, and a multiplicity-adjusted *p*-value was calculated to account for multiple comparisons with family-wise significance and confidence level at α = 0.05 (95% confidence interval) using GraphPad Software 8 (San Diego, CA, USA). The significant results for multiple comparisons between groups were marked as an asterisk as a significant difference from all in general with *p* < 0.05 (*).

## Figures and Tables

**Table 1:** Comparison of the measured contact angle of in two droplets on polymer films.

Polymer Types	Water Contact Angle, N = 10		Diiodomethane Contact Angle, N = 10	
	Top *	Bottom **	Top *	Bottom **
TPU 100%	79.5 ± 2.5°	75.6 ± 2.3°	89.3 ± 1.1°	82.4 ± 0.9°
PLDLA 100%	70.9 ± 2.1°	67.2 ± 1.7°	73.3 ± 0.9°	69.2 ± 1.0°
TPU 10% + PLDLA 90%	69.7 ± 1.1°	68.7 ± 0.8°	65.7 ± 0.7°	64.2 ± 1.2°

* On the air side; ** from the petri dish (glass surface); N; times of measurement.

**Table 2:** The mean of mass and pH changes profile for the degradable polymer films.

Time	PLDLA (100%)		TPU (100%)		TPU + PLDLA (90%/10%)	
	Mass%	pH (7.4)	Mass%	pH (7.4)	Mass%	pH (7.4)
Week 1	82.2	6.08	98.4	7.08	80.2	6.37
Week 2	76.4	6.01	96.4	6.84	76.2	6.11
Week 3	61.6	5.88	93.4	6.84	71.2	5.61
Week 4	53.4	5.80	90.8	6.71	67.4	5.79
Week 5	48.2	5.74	86.8	6.46	63.6	5.87
Week 6	44.4	5.61	82.8	6.43	57.8	5.93
Week 7	41.2	5.52	79.6	6.50	45.8	5.99

**Table 3:** The average percentage of degraded CSA implants through time.

Time	Mean ± SD
Week 1	0.122 ± 0.027
Week 2	0.162 ± 0.019
Week 3	0.198 ± 0.037
Week 4	0.218 ± 0.019
Week 5	0.356 ± 0.062
Week 6	0.896 ± 0.142
Week 7	1.074 ± 0.071

## Data Availability

Not applicable.
